# Supplementary material for: The E3 ubiquitin ligase component, Cereblon, is an evolutionarily conserved regulator of Wnt signaling
Source: Nat Commun. 2021 Sep 6;12:5263. doi: 10.1038/s41467-021-25634-z (PMC8421366; doi:10.1038/s41467-021-25634-z)
Supplement: Supplementary file 1 — Supplementary information [file 41467_2021_25634_MOESM1_ESM.pdf]

Supplementary information

The E3 Ubiquitin Ligase Component, Cereblon, is an Evolutionarily Conserved Regulator of Wnt Signaling

Chen Shen[1, 2, †], Anmada Nayak[1, †], Leif R. Neitzel[3], Amber A. Adams[4], Maya Silver-Isenstadt[3], Leah M. Sawyer[5], Hassina Benchabane[4], Huilan Wang[1], Nawat Bunnag[4], Bin Li[1], Daniel T. Wynn[1], Fan Yang[1,2], Marta Garcia-Contreras[1], Charles H. Williams[3], Sivanesan Dakshanamurthy[6], Charles C. Hong[3], Nagi G. Ayad[6, 7, 8], Anthony J. Capobianco[1, 8], Yashi Ahmed[4], Ethan Lee[5], David J. Robbins[1, 6, 8, *]

[1]Molecular Oncology Program, The DeWitt Daughtry Family Department of Surgery, Miller School of Medicine, University of Miami, Miami, FL 33136, USA.
[2]The Sheila and David Fuente Graduate Program in Cancer Biology, Miller School of Medicine, University of Miami, Miami, FL 33136, USA.
[3]Department of Medicine, University of Maryland, Baltimore, MD 21201, USA.
[4]Department of Molecular and Systems Biology and the Norris Cotton Cancer Center, Geisel School of Medicine, Dartmouth College, Hanover, NH 03755, USA.
[5]Department of Cell and Developmental Biology, Vanderbilt University, Nashville, TN 37232, USA.
[6]Department of Oncology, Lombardi Comprehensive Cancer Center, Georgetown University, Washington, DC 20057, USA.
[7]Center for Therapeutic Innovation, Department of Neurological Surgery, Miami Project to Cure Paralysis, Miller School of Medicine, University of Miami, Miami, FL 33136, USA.
[8]Sylvester Comprehensive Cancer Center, Miller School of Medicine, University of Miami, Miami, FL 33136, USA.

***Corresponding Author:**

David J. Robbins, Ph.D.

Department of Oncology,
Lombardi Comprehensive Cancer Center
Georgetown University
New Research Building, Room E520A
3970 Reservoir Road NW
Washington, DC 20057-1468
Email: dr956@georgetown.edu

[†] These authors contributed equally to this work.

Supplementary figures

Supplementary figure 1

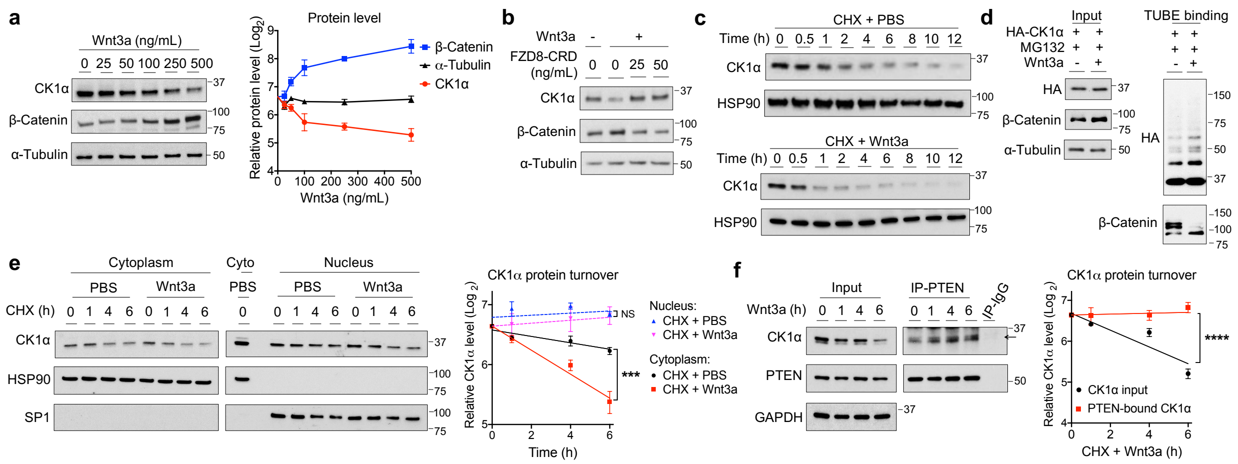

Supplementary figure 1: Wnt signaling regulates CK1α levels via ubiquitin-dependent proteasomal degradation. a. Extracts of HEK cells treated for 24 h with the indicated amounts of Wnt3a were evaluated by immunoblotting. A representative immunoblot (left panel) and a quantitation of immunoblots (mean ± SEM, n = 3 independent experiments; right panel) are shown. **b.** Extracts of HEK cells treated for 24 h with PBS, Wnt3a only, or Wnt3a and recombinant Frizzled8 cysteine-rich domain (FZD8-CRD) were evaluated by immunoblotting. A representative immunoblot (n = 3 independent experiments) is shown. **c.** Extracts of HEK cells co-treated with cycloheximide and PBS or Wnt3a for the indicated times were evaluated by immunoblotting. A representative immunoblot is shown. Fig. 1c shows the quantification of immunoblots from three such independent experiments. **d.** HEK cells were transfected with a plasmid encoding HA-tagged CK1α and subsequently treated with PBS or Wnt3a, in the presence of MG132. Extracts of these cells (left panel) were used to isolate ubiquitin modified CK1α (mean ± SEM, n = 3 independent experiments; right panel) using beads linked to tandem ubiquitin binding entities (TUBE), followed by analyses of the indicated proteins by immunoblotting. A representative immunoblot (n = 3 independent experiments) is shown. **e.** HEK cells were co-treated with cycloheximide and PBS or Wnt3a for the indicated time. Extracts of these cells were separated into cytoplasmic and nuclear fractions and evaluated by immunoblotting analysis. A representative immunoblot (left panel) and a quantification of immunoblots (mean ± SEM, n = 3 independent experiments; right panel) are shown. **f.** HEK cells were co-treated with cycloheximide and PBS or Wnt3a for the indicated time. PTEN was immunoprecipitated from cytoplasmic extracts of these cells, followed by analyses of the indicated proteins by immunoblotting. A representative immunoblot (left panel) or a quantitation of immunoblots (right panel) are shown. For quantification, CK1α levels in PTEN immunoprecipitated samples were normalized to that in IgG control, and further normalized to the level of PTEN in each immunoprecipitate. Asterisks indicate statistical significance (two-way Anova analysis, *** *p* value < 0.001, **** *p* value < 0.0001).

Supplementary figure 2

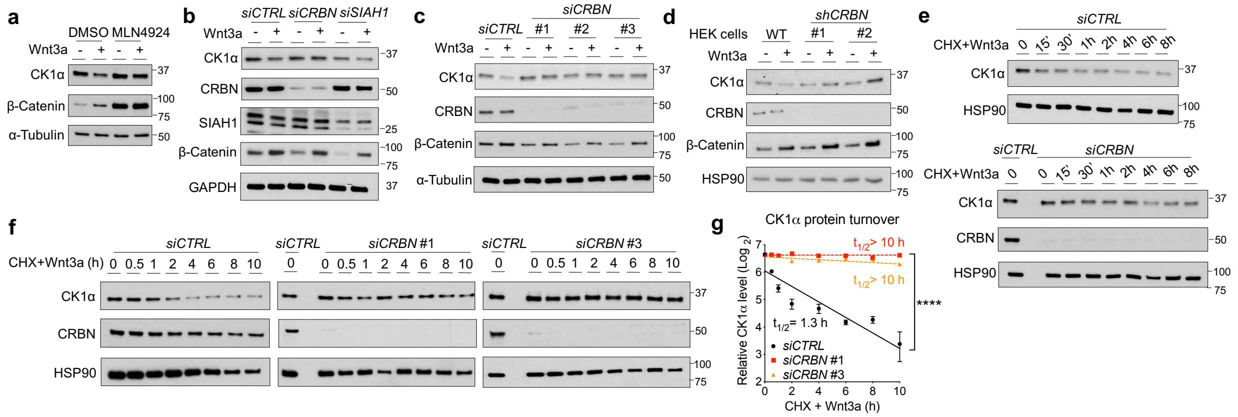

Supplementary figure 2: Wnt-induced degradation of CK1α requires CRBN, the substrate receptor of the $CRL4^{CRBN}$ E3 ubiquitin ligase complex. a. Extracts of HEK cells treated for 6 h with PBS or Wnt3a, along with DMSO or 1 μM MLN4924, were evaluated by immunoblotting. **b.** HEK cells were transfected with the indicated smart-pool siRNA and subsequently treated with PBS or Wnt3a for 24 h. Extracts of these cells were evaluated by immunoblotting. **c.** HEK cells transfected with *control* (*CTRL*) siRNA or one of three distinct *CRBN* siRNA were treated with PBS or Wnt3a for 24 h. Extracts of these cells were evaluated by immunoblotting. **d.** Wild-type (WT) HEK cells or those stably expressing one of two distinct *CRBN* shRNA were treated with PBS or Wnt3a for 24 h. Extracts of these cells were evaluated by immunoblotting. A representative immunoblot (n = 3 independent experiments) is shown in **a-d**. **e.** HEK cells were co-treated with cycloheximide and PBS or Wnt3a for the indicated time. Extracts of these cells were evaluated by immunoblotting. A representative immunoblot is shown here. Fig. 2B shows the quantification of immunoblots from three such independent experiments. **f-g.** HEK cells were transfected with the indicated siRNA and subsequently co-treated with cycloheximide and Wnt3a for the indicated time. Extracts of these cells were evaluated by immunoblotting. A representative immunoblot (**f**) and a quantification of immunoblots (mean ± SEM, n = 3 independent experiments) showing CK1α levels, normalized to that of HSP90, are shown here (**g**). Asterisks indicate statistical significance (two-way Anova analysis, **** p value < 0.0001).

Supplementary figure 3

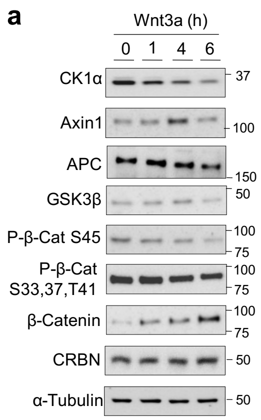

Supplementary figure 3: The β-Catenin destruction complex regulates CRBN-mediated CK1α degradation. a. Extracts of HEK cells treated with Wnt3a for various lengths of time were evaluated by immunoblotting. A representative immunoblot is shown here and the quantitation of immunoblots from three independent experiments in Fig. 3a.

Supplementary figure 4

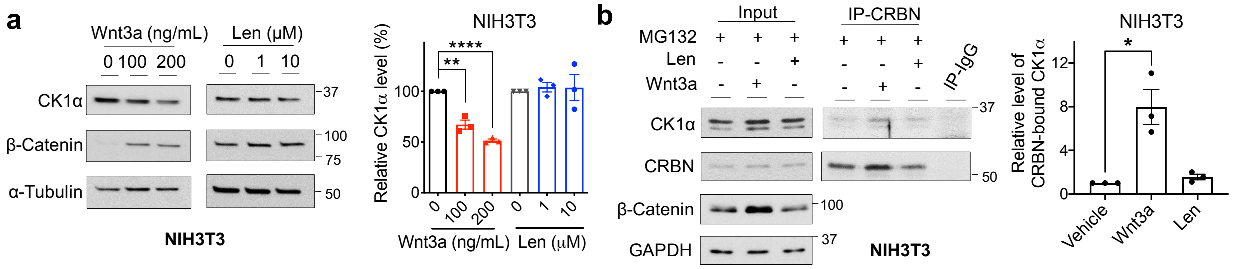

Supplementary figure 4: Wnt signaling regulates the CRBN-mediated degradation of CK1α in mouse cells. a. Extracts of mouse NIH3T3 cells treated for 24 h with different amounts of Wnt3a or lenalidomide were evaluated by immunoblotting. A representative immunoblot (left panel) or a quantitation of immunoblots (mean ± SEM, n = 3 independent experiments; right panel) are shown. **b.** CRBN was immunoprecipitated from extracts of NIH3T3 cells treated for 4 h with vehicle, Wnt3a (50 ng/mL) or lenalidomide (10 μM), in the presence of MG132, followed by analyses of the indicated proteins by immunoblotting. A representative immunoblot (left panel) or a quantitation of immunoblots (mean ± SEM, n = 3 independent experiments; right panel) are shown. For quantification, CK1α levels in CRBN immunoprecipitated samples were normalized to that in IgG control, and further normalized to the level of CRBN in each immunoprecipitate. Asterisks indicate statistical significance (two-tailed Student's *t* test, * p value < 0.05, ** p value < 0.01, **** p value < 0.00001).

Supplementary figure 5

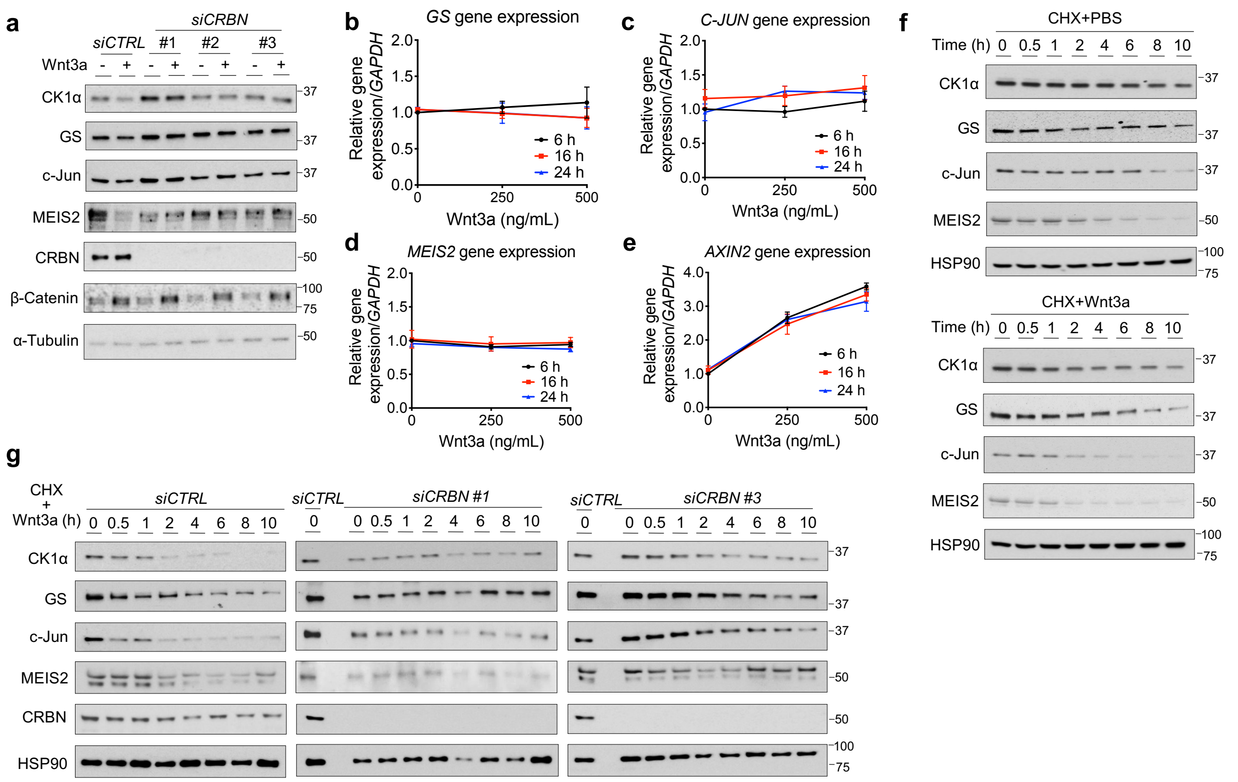

Supplementary figure 5: Wnt signaling induces the CRBN-mediated degradation of a subset of endogenous substrates. a. HEK cells were transfected with *control* (*CTRL*) siRNA or one of three distinct *CRBN* siRNA and then treated with PBS or Wnt3a for 24 h. Extracts of these cells were evaluated by immunoblotting. A representative immunoblot (n = 3 independent experiments) is shown. **b-e.** Expression of the indicated genes was quantitated in RNA from HEK cells treated with PBS or Wnt3a for various lengths of time, using quantitative RT-PCR. A quantification of gene expression (mean ± SEM, n = 3 independent experiments) is shown. **f.** HEK cells were co-treated with cycloheximide and PBS or Wnt3a for the indicated time. Extracts of these cells were evaluated by immunoblotting, and a representative immunoblot is shown. The quantification of immunoblots from three independent experiments is shown in Fig. 5b-d. **g.** HEK cells were transfected with *control* (*CTRL*) siRNA or one of two distinct *CRBN* siRNA and co-treated with cycloheximide and Wnt3a for the indicated time. Extracts of these cells were evaluated by immunoblotting, and a representative immunoblot is shown. The quantification of immunoblots from three independent experiments is shown in Fig. 5e-g.

Supplementary figure 6

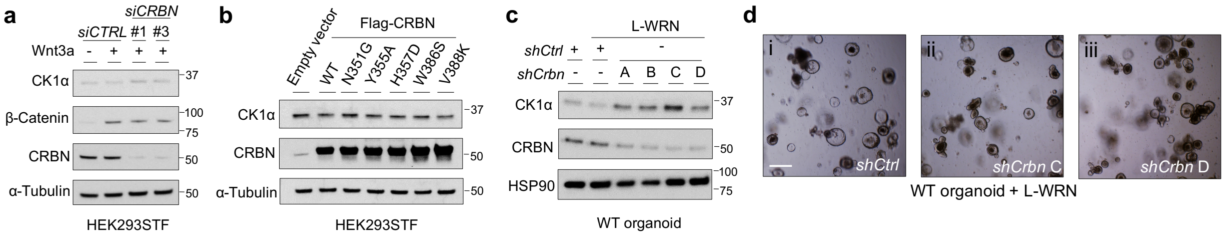

Supplementary figure 6: CRBN is a positive regulator of Wnt activity. a. HEK293STF cells were transfected with *control* (*CTRL*) siRNA or one of two distinct *CRBN* siRNA along with PBS or Wnt3a (100 ng/mL) treatment for 48 h. Extracts of these cells were evaluated by immunoblotting, and a representative immunoblot is shown. The quantification of Wnt reporter activity from three independent experiments is shown in Fig. 6a. **b.** HEK293STF cells were transfected with a control plasmid or a plasmid encoding wild-type (WT) Flag-tagged CRBN, or the indicated Flag-tagged CRBN mutants for 48h. Extracts of these cells were evaluated by immunoblotting, and a representative immunoblot is shown. The quantification of Wnt reporter activity of three independent experiments is shown in Fig. 6b. **c-d.** Mouse intestinal organoids were infected with *Control* (*Ctrl*) or distinct *Crbn* shRNA and cultured in basal media or 25% L-WRN conditioned media for 5 days. (**c**) Organoid lysates were used to immunoblot the indicated proteins and a representative immunoblot (n = 3 independent experiments) is shown. The corresponding state of organoid differentiation was analyzed and shown in Fig. 6c. (**d**) Low magnification, representative images of multiple mouse intestinal organoids from each treatment group used in Fig. 6c (n = 3 independent experiments) are shown here. Scale bar = 200 μm.

Supplementary figure 7

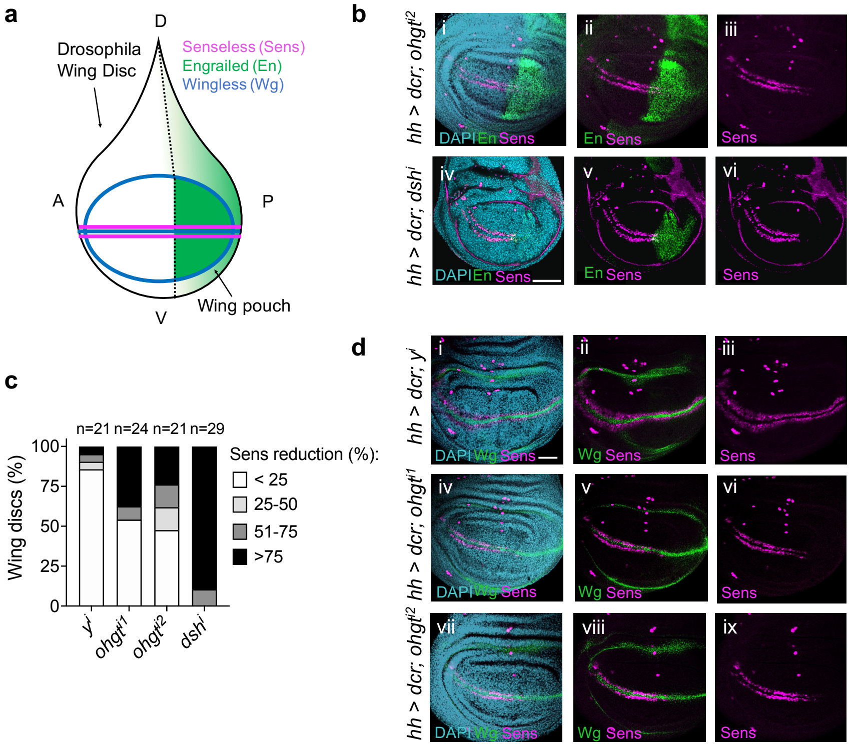

Supplementary figure 7: CRBN modulates Wnt function in Drosophila. a. A schematic of a *Drosophila melanogaster* wing imaginal disc. A: anterior; P: posterior; D: dorsal; V: ventral. **b.** Representative confocal images showing the level of a Wg/Wnt biomarker Senseless (Sens, magenta) after RNAi-mediated knockdown of (i-iii) *ohgata* (*ohgt/crbn*) (n = 43 independent Drosophila wing discs) or (iv-vi) *dishevelled (dsh)* (n = 29 independent Drosophila wing discs), driven by a *hh-Gal4* driver in the posterior compartment of third instar wing imaginal discs. The region in which *hh-Gal4* drives expression is indicated by Engrailed (En) in green. DAPI staining (blue) is used to mark the wing disc. *hh*: *hedgehog*; *dcr*: *dicer*. Scale bar = 50 µm. Representative images of wing discs with *yellow* (*y*) control or *ohgata* (*ohgt/crbn*) knockdown by a separate RNAi are shown in Fig. 7b. **c.** A quantification of the percent of wing discs with differential levels of Sens reduction upon the indicated gene knockdown. The length of Sens remaining in the posterior region was measured and normalized to the total length of the posterior compartment. **d.** Representative confocal images showing the level of a Wg/Wnt biomarker Senseless (Sens, magenta) and a Notch biomarker Wingless (Wg, green) after RNAi-mediated knockdown of (i-iii) *y* control (n = 42 independent Drosophila wing discs) or (iv-ix) *ohgt* (n = 47 or 43 independent Drosophila wing discs), driven by a *hh-Gal4* driver in the posterior compartment of third instar wing imaginal discs. DAPI staining (blue) is used to mark the wing disc. *hh*: *hedgehog*; *dcr*: *dicer*. Scale bar = 50 µm.

Supplementary figure 8

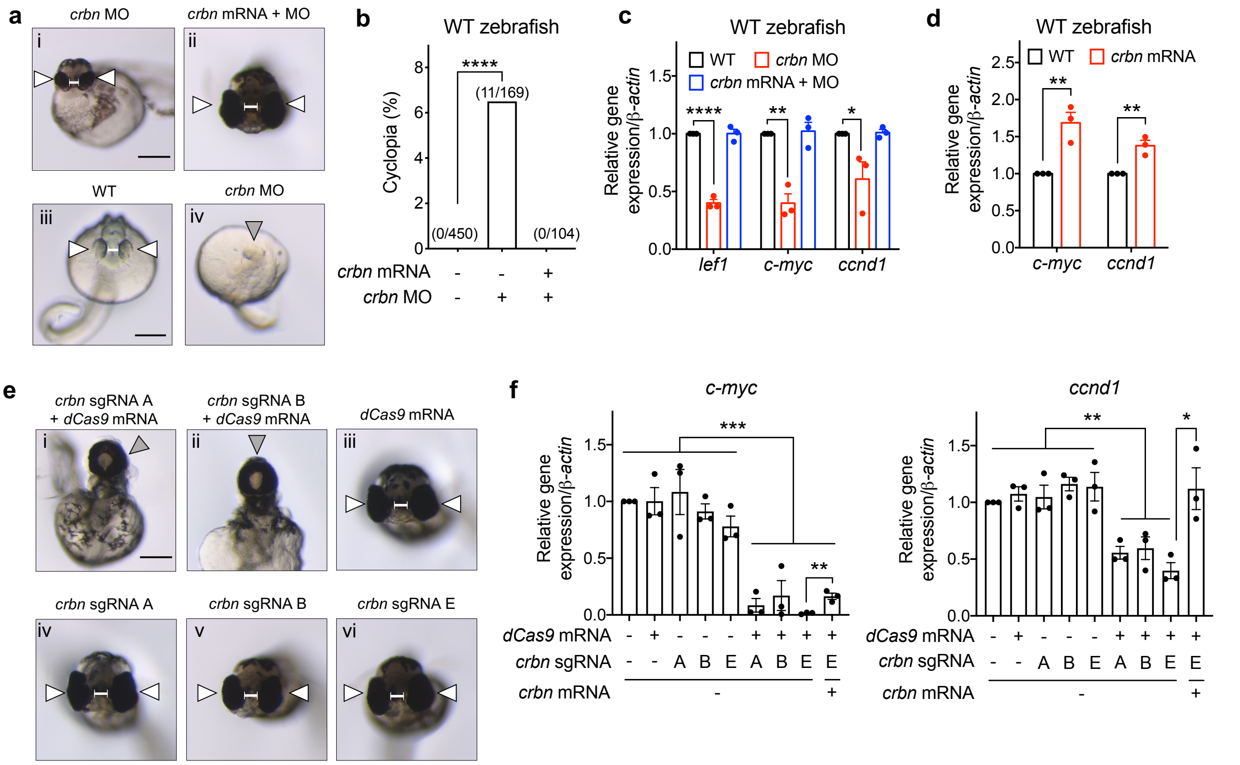

Supplementary figure 8: CRBN modulates Wnt function in zebrafish. a. The transverse views of 2 dpf WT zebrafish injected with (i) *crbn* morpholino (MO) alone or (ii) that along with *crbn* mRNA or (iii) 1 dpf WT zebrafish or (iv) that injected with *crbn* MO. Representative images of 2 dpf WT zebrafish and that with other treatments are shown in Fig. 8b. Scale bar = 200 µm. White arrows indicate eyes, the grey arrow indicates merged eyes, and the balbis indicates the distance between eyes. **b.** Quantifications of the indicated eye phenotypes (2 dpf) are shown. Asterisks indicate statistical significance (Fisher's exact test, **** p value < 0.0001). **c-d.** RNA extracts from the indicated zebrafish (20 hpf) were used to determine the expression of three Wnt target genes using quantitative RT-PCR. A quantification of gene expression (mean ± SEM, n = 3 independent pools of zebrafish embryo) is shown. Asterisks indicate statistical significance (two-tailed Student's t test, * p value < 0.05, ** p value < 0.01, **** p value < 0.0001). *ccnd1*: *cyclin D1*; *lef1*: *lymphoid enhancer-binding factor 1*. *lef1* expression of embryos in **d** is shown in Fig. 8d. **e.** The transverse views of 2 dpf WT zebrafish injected with one of three distinct *crbn* guide RNAs (sgRNA) or/and *dCas9* mRNA. Scale bar = 200 µm. White arrows indicate eyes, the grey arrow indicates merged eyes, and the balbis indicates the distance between eyes. **f.** RNA extracts from the indicated zebrafish (20 hpf) were used to determine the expression of three Wnt target genes using quantitative RT-PCR. A quantification of gene expression (mean ± SEM, n = 3 independent pools of zebrafish embryo) is shown. *lef1* expression of these embryos is shown in Fig. 8f. Asterisks indicate statistical significance (one-way Anova analysis or two-tailed Student's t test, * p value < 0.05, ** p value < 0.01, *** p value < 0.001).

Supplementary figure 9

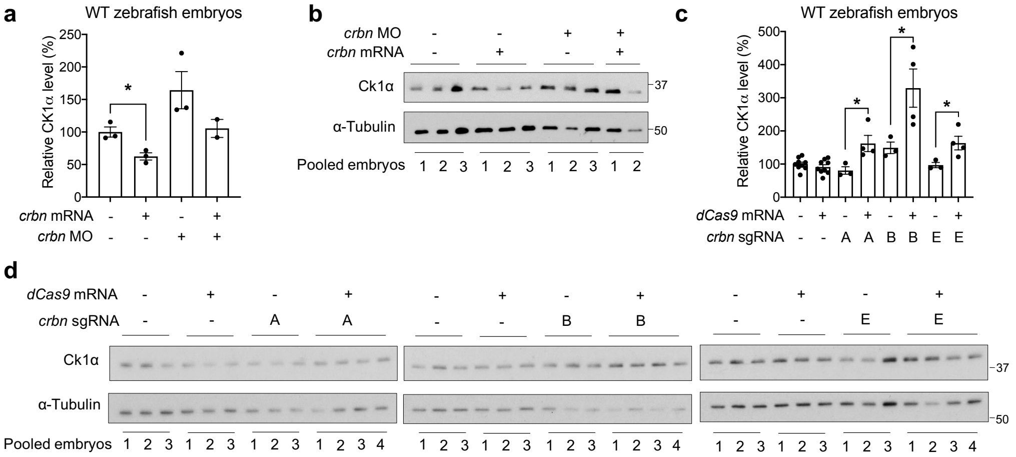

Supplementary figure 9: CRBN modulates Ck1α level in zebrafish. a-b. Protein extracts of pooled 15 hpf WT zebrafish embryos and those injected with *crbn* mRNA or/and *crbn* MO were used for immunoblotting analysis. A representative immunoblot (b) and a quantification of the immunoblot (a) are shown (n = 3, 3, 3, or 2 independent pooled zebrafish embryos). **c-d.** Protein extracts of pooled 15 hpf WT zebrafish embryos and those injected with *crbn* sgRNA or/and *dCas9* mRNA were used for immunoblotting analysis. A representative immunoblot (d) and a quantification of the immunoblot (c) are shown (n = 3, 3, 3, 4, 3, 4, 3, or 4 samples of independent pooled zebrafish embryos). Asterisks indicate statistical significance (two-tailed Student's *t* test, * p value < 0.05).

Supplementary tables

Supplementary table 1: List of primers for qRT-PCR used in this study.

**Homo sapiens gene**	**Source**	**Catalog #**
CK1α	Invitrogen	Hs00793391
AXIN2	Invitrogen	Hs00610344
GAPDH	Invitrogen	Hs02758991
TBP	Invitrogen	Hs00427620
C-JUN	Invitrogen	Hs01103582
GS	Invitrogen	Hs00365928
MEIS2	Invitrogen	Hs00542638
***Danio rerio* gene**	**Sequence 5'-3'**	
β-actin	Forward: CGAGCTGTCTTCCCATCCA<br>Reverse: TCACCAACGTAGCTGTCTTTCTG	
lef1	Forward: GAGGGAAAAGATCCAGGAAC<br>Reverse: AGGTTGAGAAGTCTAGCAGG	
ccnd1	Forward: GCCAAACTGCCTATACATCAG<br>Reverse: TGTCGGTGCTTTTCAGGTAC	
c-myc	Forward: TAACAGCTCCAGCAGCAGTG<br>Reverse: GCTTCAAAACTAGGGGACTG	

Supplemental table 2: List of primary antibodies used in this study.

**Antigen**	**Source**	**Catalog #**	**Dilution**
CK1α	Abcam	ab108296	1:1000
SIAH1	Abcam	ab2237	1:250
Axin1	Cell Signaling Technology	2087	1:1000
APC	Cell Signaling Technology	2504	1:1000
GSK3β	Cell Signaling Technology	9315	1:1000
p-β-Catenin S45	Cell Signaling Technology	9564	1:1000
p-β-Catenin S33,37,T41	Cell Signaling Technology	9561	1:1000
β-Catenin	Cell Signaling Technology	9562	1:1000
c-Myc	Cell Signaling Technology	5605	1:5000
GAPDH	Cell Signaling Technology	8884	1:5000
c-Jun	Cell Signaling Technology	9165	1:5000
PTEN	Cell Signaling Technology	9552	1:5000
α-Tubulin	Cell Signaling Technology	9099	1:5000
α-Tubulin	Millipore	T9199	1:5000
GS	Millipore	MAB302	1:1000
Ubiquitin	Santa Cruz Biotechnology	sc-8017	1:500
HSP90	Santa Cruz Biotechnology	sc-13119	1:5000
P62	BD Biosciences	610832	1:1000
CRBN	Novus Biologicals	NBP1-91810	1:5000
MEIS2	Novus Biologicals	H00004212-M01	1:250
Flag	Sigma	F7425	1:5000
