# Supplementary material for: The E3 ubiquitin ligase component, Cereblon, is an evolutionarily conserved regulator of Wnt signaling
Source: Nat Commun. 2021 Sep 6;12:5263. doi: 10.1038/s41467-021-25634-z (PMC8421366; doi:10.1038/s41467-021-25634-z)
Supplement: Supplementary file 3 — Reporting Summary [file 41467_2021_25634_MOESM3_ESM.pdf]

nature research

Corresponding author(s): David J. Robbins

Last updated by author(s): Jul 21, 2021

Reporting Summary

Nature Research wishes to improve the reproducibility of the work that we publish. This form provides structure for consistency and transparency in reporting. For further information on Nature Research policies, see our Editorial Policies and the Editorial Policy Checklist.

Statistics

For all statistical analyses, confirm that the following items are present in the figure legend, table legend, main text, or Methods section.

n/a	Confirmed	
☐	☒	The exact sample size (*n*) for each experimental group/condition, given as a discrete number and unit of measurement
☐	☒	A statement on whether measurements were taken from distinct samples or whether the same sample was measured repeatedly
☐	☒	The statistical test(s) used AND whether they are one- or two-sided<br>*Only common tests should be described solely by name; describe more complex techniques in the Methods section.*
☐	☒	A description of all covariates tested
☒	☐	A description of any assumptions or corrections, such as tests of normality and adjustment for multiple comparisons
☐	☒	A full description of the statistical parameters including central tendency (e.g. means) or other basic estimates (e.g. regression coefficient) AND variation (e.g. standard deviation) or associated estimates of uncertainty (e.g. confidence intervals)
☐	☒	For null hypothesis testing, the test statistic (e.g. *F*, *t*, *r*) with confidence intervals, effect sizes, degrees of freedom and *P* value noted<br>*Give P values as exact values whenever suitable.*
☒	☐	For Bayesian analysis, information on the choice of priors and Markov chain Monte Carlo settings
☒	☐	For hierarchical and complex designs, identification of the appropriate level for tests and full reporting of outcomes
☒	☐	Estimates of effect sizes (e.g. Cohen's *d*, Pearson's *r*), indicating how they were calculated

Our web collection on statistics for biologists contains articles on many of the points above.

Software and code

Policy information about availability of computer code

Data collection	Quantitative PCR data of HEK293T cells were collected by Bio-Rad CFX manager 3.1 and that of zebrafish embryos by Quant Studio 5 real-time PCR systems (Applied Systems). Fluorescence signal were collected by a Veritas microplate reader. Images of organoids were obtained using an Olympus IX51 inverted fluorescence microscope and Olympus CellSens software. Images of drosophila wing disc were obtained using a Nikon A1RSi confocal microscope. Images of zebrafish embryos were obtained using a Zeiss Stemi 2000-CS microscope with an Olympus DP72 camera.
Data analysis	Two-tailed student's t-test, Two-way ANOVA, and Fisher's exact test were done using Prism GraphPad 9. Quantitative PCR data were analyzed by Biorad CFX manager 3.1. Immunoblots were analyzed using ImageJ.

For manuscripts utilizing custom algorithms or software that are central to the research but not yet described in published literature, software must be made available to editors and reviewers. We strongly encourage code deposition in a community repository (e.g. GitHub). See the Nature Research guidelines for submitting code & software for further information.

Data

Policy information about availability of data

All manuscripts must include a data availability statement. This statement should provide the following information, where applicable:

- Accession codes, unique identifiers, or web links for publicly available datasets
- A list of figures that have associated raw data
- A description of any restrictions on data availability

All data supporting the findings of this study are available in the paper and the Supplementary information file. Raw data and original gel images are included in the Source Data file. All other relevant data are available from the authors upon reasonable request.

Field-specific reporting

Please select the one below that is the best fit for your research. If you are not sure, read the appropriate sections before making your selection.

☒ Life sciences ☐ Behavioural & social sciences ☐ Ecological, evolutionary & environmental sciences

For a reference copy of the document with all sections, see nature.com/documents/nr-reporting-summary-flat.pdf

Life sciences study design

All studies must disclose on these points even when the disclosure is negative.

Sample size

Ex vivo experiments were performed at least in triplicate and repeated three times. Nine measures per condition yield 84% power to detect a standardized difference (Δ group averages/common SD) of 1.5 based on a two-sample t-test with two-sided α=0.05.

For drosophila studies, at least 21 samples were included in each group. For zebrafish studies, at least 31 samples were included in each group. Sample size was determined consistently with previous publications in the field. These sample sizes ensure adequate statistical power as well as reproducibility of our assays.

Data exclusions

In zebrafish studies, embryos with severe edema and dead embryos were removed from the sample sets.

Replication

Each experiment in this study is successfully repeated for at least 3 times.

Randomization

Drosophila and zebrafish used in this study were randomly separated into each groups. For experiment using cell-lines or organoids, no specific passage of cells was required and cells were randomly separated into groups.

Blinding

Researchers were not blinded during this study to ensure data are correctly collected from each treatment group. In addition, to avoid bias, the majority of our data were analyzed in a quantifiable fashion, followed by determination of statistical significance.

Reporting for specific materials, systems and methods

We require information from authors about some types of materials, experimental systems and methods used in many studies. Here, indicate whether each material, system or method listed is relevant to your study. If you are not sure if a list item applies to your research, read the appropriate section before selecting a response.

Materials & experimental systems

n/a	Involved in the study	
☐	☒	Antibodies
☐	☒	Eukaryotic cell lines
☒	☐	Palaeontology and archaeology
☐	☒	Animals and other organisms
☒	☐	Human research participants
☒	☐	Clinical data
☒	☐	Dual use research of concern

Methods

n/a	Involved in the study	
☒	☐	ChIP-seq
☒	☐	Flow cytometry
☒	☐	MRI-based neuroimaging

Antibodies

Antibodies used

Immunoblotting: The primary antibodies used were CK1a (ab108296) and SIAH1 (ab2237) from Abcam, Axin1 (2087), APC (2504), GSK3B (9315), phosphorylated B-Catenin S45 (9564), phosphorylated B-Catenin S33, 37, T41 (9561), B-Catenin (9562), c-Myc (5605), GAPDH (8884), c-Jun (9165), PTEN (9552) and A-Tubulin (9099) from Cell Signaling Technology, A-Tubulin (T6199) and GS (MAB302) from Millipore, Ubiquitin (sc-8017) and HSP90 (sc-13119) from Santa Cruz Biotechnology, P62 (610832) from BD Biosciences, CRBN (NBP1-91810) and MEIS2 (H00004212-M01) from Novus Biologicals, and Flag (F7425) from Sigma. The secondary antibodies used were HRP-conjugated donkey anti-mouse or anti-rabbit (715-035-150 or 711-035-152), or rabbit anti-goat IgG (305-025-045) from Jackson ImmunoResearch.

Immunoprecipitation: The antibodies used were CK1a (Abcam ab206652), CRBN (Abcam ab68763 or Cell signaling technology 71810) and PTEN (Santa Cruz Biotechnology sc-393186).

Validation

The validation of antibodies used was performed by the individual companies and multiple publications:
CK1a (Abcam ab108296): https://www.abcam.com/csnk1a1--csnk1a1l-antibody-epr19612-ab108296.html;
SIAH1: https://www.abcam.com/siah1-antibody-ab2237.html;
Axin1: https://www.cellsignal.com/datasheet.jsp?productId=2087&images=1;
APC: https://www.cellsignal.com/datasheet.jsp?productId=2504&images=1;
GSK3B: https://www.cellsignal.com/datasheet.jsp?productId=9315&images=1;
p-B-Cat S45: https://www.cellsignal.com/datasheet.jsp?productId=9564&images=1;

p-B-Cat S33,37,T41: https://www.cellsignal.com/datasheet.jsp?productId=9561&images=1;
B-Catenin: https://www.cellsignal.com/datasheet.jsp?productId=9562&images=1;
c-Myc: https://www.cellsignal.com/datasheet.jsp?productId=5605&images=1;
GAPDH: https://www.cellsignal.com/datasheet.jsp?productId=8884&images=1;
c-Jun: https://www.cellsignal.com/datasheet.jsp?productId=9165&images=1;
PTEN (CST 9552): https://www.cellsignal.com/datasheet.jsp?productId=9552&images=1;
A-Tubulin (CST 9099): https://www.cellsignal.com/datasheet.jsp?productId=9099&images=1;
A-Tubulin (Millipore T6199): https://www.sigmaaldrich.com/deepweb/assets/sigmaaldrich/product/documents/312/421/t6199dat.pdf;
GS: https://www.emdmillipore.com/US/en/product/Anti-Glutamine-Synthetase-Antibody-clone-GS-6,MM_NF-MAB302;
Ubiquitin: https://datasheets.scbt.com/sc-8017.pdf;
HSP90: https://datasheets.scbt.com/sc-13119.pdf;
CRBN (Novus NBP1-91810): https://www.novusbio.com/PDFs/NBP1-91810.pdf;
MEIS2: https://www.novusbio.com/PDFs/H00004212-M01.pdf;
Flag: https://www.sigmaaldrich.com/deepweb/assets/sigmaaldrich/product/documents/799/916/f7425dat.pdf;
CRBN (CST 71810): https://www.cellsignal.com/datasheet.jsp?productId=71810&images=1;
PTEN (SCBT sc-393186): https://datasheets.scbt.com/sc-393186.pdf.

CRBN (Abcam ab68763) in immunoprecipitation was validated by a previous publication (Kroenke et al. Nature, 2015).

Eukaryotic cell lines

Policy information about cell lines

Cell line source(s)	HEK293T (HEK), HEK293STF, NIH3T3 and L-WRN cell lines used in this study were purchased from ATCC.
Authentication	All cell lines used in this study were routinely authenticated by ATCC by STR profiling.
Mycoplasma contamination	No cell line was tested with mycoplasma contamination.
Commonly misidentified lines (See ICLAC register)	To our knowledge, there were no commonly misidentified lines used in this study.

Animals and other organisms

Policy information about studies involving animals; ARRIVE guidelines recommended for reporting animal research

Laboratory animals	Wild-type NHGRI zebrafish zygotes (1 cell) were injected with crbn constructs used in this study.<br><br>Drosophila RNAi lines (ohgti1: Vienna Drosophila Resource Center (VDRC) #110809 and oghti2: VDRC #40486 and yi: VDRC #106068 and dshi: Bloomington Drosophila Stock Center #31306) were expressed using the hh-Gal4 driver in conjunction with UAS-Dcr-2 (Bloomington Drosophila Stock Center #25757) in the wing discs of third instar male larval .
Wild animals	No wild animal was used in this study.
Field-collected samples	No field-collected samples were used in this study.
Ethics oversight	Zebrafish studies were performed following the animal protocol approved by University of Maryland's Institutional Animal Care and Use Committee.

Note that full information on the approval of the study protocol must also be provided in the manuscript.
